# Assessing the Reliability of Template-Based Clustering for Tractography in Healthy Human Adults

**DOI:** 10.3389/fninf.2022.777853

**Published:** 2022-02-17

**Authors:** Jason Kai, Ali R. Khan

**Affiliations:** ^1^Department of Medical Biophysics, Schulich School of Medicine and Dentistry, The University of Western Ontario, London, ON, Canada; ^2^Centre for Functional and Metabolic Mapping, Robarts Research Institute, The University of Western Ontario, London, ON, Canada

**Keywords:** tractography, diffusion MRI, white matter, template clustering, reliability

## Abstract

Tractography is a non-invasive technique to investigate the brain’s structural pathways (also referred to as tracts) that connect different brain regions. A commonly used approach for identifying tracts is with template-based clustering, where unsupervised clustering is first performed on a template in order to label corresponding tracts in unseen data. However, the reliability of this approach has not been extensively studied. Here, an investigation into template-based clustering reliability was performed, assessing the output from two datasets: Human Connectome Project (HCP) and MyConnectome project. The effect of intersubject variability on template-based clustering reliability was investigated, as well as the reliability of both deep and superficial white matter tracts. Identified tracts were evaluated by assessing Euclidean distances from a dataset-specific tract average centroid, the volumetric overlap across corresponding tracts, and along-tract agreement of quantitative values. Further, two template-based techniques were employed to evaluate the reliability of different clustering approaches. Reliability assessment can increase the confidence of a tract identifying technique in future applications to study pathways of interest. The two different template-based approaches exhibited similar reliability for identifying both deep white matter tracts and the superficial white matter.

## Introduction

The brain consists of numerous regions connected together by axonal bundles which form the structural pathways (also referred to as tracts) (Sarwar et al., 2019; Sotiropoulos and Zalesky, 2019) of a highly connected network that enables function and cognition (Mesulam, 1998; Klingberg et al., 1999; Jbabdi et al., 2015; Filley and Fields, 2016). Although the gold standard for investigating structural connectivity are chemical tracers, these techniques are invasive and performed only in animal studies and post-mortem samples (Jbabdi et al., 2015; Sotiropoulos and Zalesky, 2019). Alternatively, the brain’s connectivity can be studied non-invasively, *in vivo* with diffusion magnetic resonance imaging (dMRI). Briefly, dMRI acquires directionally sensitive information about the diffusion of water molecules (Conturo et al., 1999), which preferentially diffuses in parallel to the axonal trajectory (Bammer, 2003). Using information from dMRI, an estimation of the pathway trajectories can be reconstructed as a streamline with tractography by (1) estimating the diffusion orientation within all image voxels and (2) following along an orientation voxel-to-voxel until a termination criterion is met (Sotiropoulos and Zalesky, 2019). Past studies have examined how long-range tracts connecting distant brain regions (Bernasconi et al., 2004; Govindan et al., 2008; Riley et al., 2010; Wu et al., 2018; Zhang et al., 2018a) and short-range, “U”-shaped tracts comprising the superficial white matter (Yendiki et al., 2011; Guevara et al., 2012, 2017; Tunç et al., 2014) are affected in neurological or psychiatric disorders. Furthermore, quantitative differences identified in the structural pathways of patient groups have been correlated with clinical symptoms (Riley et al., 2010). An understanding of how tracts are affected in patient cohorts could provide key insights for diagnosis and improve treatment.

To identify different tracts from tractography, either manual or automated techniques can be employed. Manual techniques require users to place inclusion and exclusion regions of interest (ROI) to extract tracts for further investigation, a laborious and time-consuming task requiring anatomical knowledge with results that can vary between different users or sessions (Tunç et al., 2014). An alternative to manual ROIs is to leverage atlas-based ROIs, which require an adequate registration with an individual’s data (Yendiki et al., 2011; Guevara et al., 2012, 2017; Tunç et al., 2014) to automate identification of ROIs to extract tracts. However, this still requires anatomical knowledge to select the ROIs needed to isolate each tract of interest. One automated alternative that does not require ROIs is TRACULA, which instead uses information of surrounding anatomical structures to identify tracts (Yendiki et al., 2011). While the described approaches can aid in tract identification with a high degree of anatomical accuracy, they rely on and are limited by *a priori* knowledge (Schilling et al., 2020). Other automated techniques attempt to identify tracts with a data-driven approach, employing unsupervised clustering algorithms that commonly rely on the similarity of streamline trajectories (Tunç et al., 2014). These clustering approaches, which are not dependent on a priori knowledge, may identify previously unnamed or unidentified tracts and have been shown to produce known pathways with high confidence (Voineskos et al., 2009; Tunç et al., 2014). To identify the same tracts across individuals, a labeled template is first created from clustering together streamlines by similarity. Clusters are arbitrarily labeled for identification with no anatomical reference. The template is then registered to different individuals to identify similar tracts in a template-based clustering approach. While automated clustering techniques may include incomplete or false positive streamlines (Tunç et al., 2014), user biases from manual intervention are avoided (Voineskos et al., 2009). A number of studies have taken a template-based clustering approach to identify tracts of interest, including O’Donnell and Westin (2007), Guevara et al. (2012, 2017), Tunç et al. (2014), Román et al. (2017), Garyfallidis et al. (2018), and Zhang et al. (2018b) to name a few. Although both template-based and atlas-based approaches have been used to identify tracts, the primary difference between the two approaches is the use of predefined ROIs from anatomical atlases and *a priori* anatomical knowledge for atlas-based approaches to identify and name tracts, while template-based approaches uses the similarity of tract features to identify corresponding tracts.

Reliability of template-based clustering approaches, that is, the ability to extract corresponding tracts successfully when applying the same methodology to multiple scans of the same subject or multiple subjects, is critically important and increases confidence applying the same approach to study of tracts of interest. In the previously mentioned studies, Tunç et al. (2014) used a template created from the same individuals studied, while O’Donnell and Westin (2007) and Garyfallidis et al. (2018) highlighted clustering techniques to identify tracts. Zhang et al. (2019) used the same atlas previously developed by their group to compare the performance of template-based clustering against an ROI-based technique using three different test-retest datasets across varying age groups, highlighting the benefits of a template-based approach. Guevara et al. (2012) proposed a template-based clustering method to extract “U”-shaped tracts and created a superficial white matter atlas. Later, Guevara et al. (2017) expanded the technique to examine “U”-shaped tract reliability and produce a new superficial white matter atlas containing tracts present in at least 30% of the subjects within the dataset, producing the most common “U”-shaped tracts across those individuals. Despite the use of these techniques in studies of structural connectivity, in-depth comparisons have yet to be performed to evaluate the parallels between different template-based approaches. Further, the effect of individual differences on reliably identifying tracts has not yet been examined. Lastly, an investigation of the use of template-based approaches to reliably identify and examine superficial white matter, where individual differences can be found due to varying cortical folding, has yet to be extensively studied.

In this work, we evaluate the reliability of template-based clustering of whole-brain tractography applied to both different subjects and within a single subject using two clustering approaches—spectral clustering and QuickBundles. Both clustering approaches are applied to two open source datasets of healthy individuals: (1) Human Connectome Project, and (2) MyConnectome Project, examining all identified tracts. While the goal of tract identification is to enable investigations of tracts and study changes in patient populations, reliable identification is a non-trivial task, even amongst healthy individuals. Pathology can complicate the ability to quantify reliability by introducing heterogenous changes to the structural connectivity in different individuals. First, we assess the reliability of template-based clustering of whole-brain tractography. We follow-up by separately assessing the reliability of clustering short-range, “U”-shaped pathways, where greater intersubject variability is expected than in long-range tracts due to differing cortical folding patterns and different clustering parameters and constraints are required.

## Materials and Methods

All processing and analysis was performed within containerized environments on high performance compute clusters hosted by Compute Canada. Environments contained installations of Nipype (Gorgolewski et al., 2011), for creating reproducible pipelines, and MRtrix3 (Tournier et al., 2019) for tractography processing^1^ and implementation of spectral clustering.^2^ Additionally, QuickBundles (Garyfallidis et al., 2012) clustering, as implemented within the DIPY library (Garyfallidis et al., 2014), was also used as a secondary clustering technique.

**FIGURE 1:**
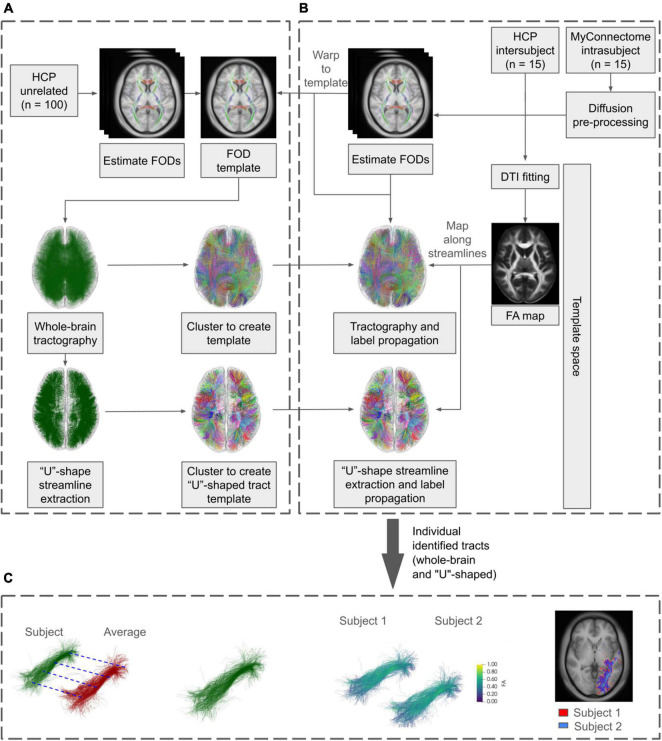
General diffusion processing workflow using minimally preprocessed HCP and unprocessed MyConnectome data. **(A)** HCP unrelated subjects were used to create the population-based FOD template. Whole-brain and “U”-shaped tractography was created from the FOD template and streamlines were assigned labels via clustering, creating labeled tractography templates. A subset of each identified tract is extracted and used to propagate labels to the analysis datasets. **(B)** MyConnectome data was first preprocessed using in-house pipelines. Together with minimally preprocessed the HCP dataset, individual FODs were computed and warped to the previously created template. Tractography was performed for each subject/session in the template space. Additionally, DTI fitting was performed and mapped along the generated streamlines. Labels from the tractography template were propagated to subject/session’s tractography. **(C)** Analysis was performed on the identified tracts, evaluating Euclidean distances and tract overlaps both within each dataset and against the labeled template. In addition, streamline counts and along-tract agreement of FA were assessed within each dataset.

An overview of the general workflow applied is shown in Figure 1. Briefly, a labeled population template was created from minimally preprocessed data and template-based clustering was applied to two separate datasets. Subsequent analysis was performed on the identified tracts, assessing the metrics across identified tracts within each dataset.

### Data Acquisition and Pre-processing

#### Template Dataset

Minimally pre-processed dMRI data, as described in Glasser et al. (2013), from the HCP1200 release of the Human Connectome Project (HCP) (Van Essen et al., 2013) of 100 unrelated subjects (46 male, 54 female; aged 22–35), here-on defined as HCPUR100, was used to first create a clustered tractography template. Structural T1w data of these subjects were also used to create an anatomical template for cortical parcellation and lobular assignment lobes via FreeSurfer (Fischl et al., 2004). dMRI data was acquired on a customized Siemens Skyra 3T scanner (Van Essen et al., 2012; Sotiropoulos et al., 2013) with the following scanning parameters: repetition time/echo time (TR/TE) = 5520/89.50 ms; resolution = 1.25 × 1.25 ×1.25 mm^3^; *b*-values = 1,000, 2,000, 3,000 s/mm^2^ (90 directions each) with 18 *b*-value = 0 s/mm^2^ images. Full acquisition details can be found in the HCP1200 subject reference manual.^3^

#### Analysis Datasets

Two separate datasets from HCP and the MyConnectome Project (Poldrack et al., 2015) were used to assess reliability of template-based clustering, here-on referred to as the analysis datasets. From the HCP analysis dataset, an additional 15 subjects (8 male, 7 female; aged 22–35) were randomly selected from the HCP1200 release for analysis, matching the number of available sessions available in the MyConnecotome analysis dataset. Acquisition parameters were previously described in the template dataset subsection.

From the MyConnectome Project, a single male subject (aged 45 at onset of data acquisition), scanned on multiple occurrences acquired over a 3 year period as a part of the MyConnectome Project was used for analysis. Data acquisition was performed on a separate Siemens Skyra 3T scanner. Out of 94 production sessions, 15 had dMRI acquisitions available for assessment, excluding a follow-up session acquired on a separate imaging system. Scanning parameters are as follows: repetition time/echo time (TR/TE) = 5,000/108 ms; resolution = 1.74 × 1.74 × 1.7 mm^3^; *b*-values = 1,000, 2,000 s/mm^2^ (30 directions each) with 4 *b*-value = 0 s/mm^2^ images. Detailed information on data collection can be found in the study protocol.^4^ Using an in-house developed pipeline, prepdwi (Khan et al., 2021), dMRI acquisitions were pre-processed. Briefly, principal component analysis based denoising (Veraart et al., 2016a,b) was performed followed by unringing of the dMRI data to minimize the effects of Gibbs ringing (Kellner et al., 2016). Afterward, FSL’s topup (Andersson et al., 2003; Smith et al., 2004) and eddy (Andersson and Sotiropoulos, 2016) were applied to correct for distortions induced by susceptibility, eddy currents, and subject motion.

### Tractography Processing

The following sections describe the processing steps performed for generated tractography, including clustering and assignment of labels to streamlines.

#### Fiber Orientation Distribution

A fiber orientation distribution (FOD) template was created with the HCPUR100 using the MRtrix3 software suite (Tournier et al., 2019). Briefly, a tissue-specific (white matter, gray matter, and cerebrospinal fluid) response function was estimated for each HCPUR100 subject using the Dhollander algorithm (Dhollander et al., 2016), before averaging the computed response functions. Utilizing the average response function, FODs were estimated for each HCPUR100 subject using a multi-shell, multi-tissue constrained spherical deconvolution (MSMT-CSD) algorithm (Jeurissen et al., 2014) and normalized with a multi-tissue informed log-domain intensity normalization (Raffelt et al., 2017). Normalized FODs were transformed using a multi-resolution pyramid structure to create an FOD template (Raffelt et al., 2011). Registrations were optimized with six iterations of rigid and affine transformations each, and 15 iterations of non-linear transformation. The FOD template was utilized to transform analysis data to a common midway space (defined as the template space).

Similar steps were taken to compute FODs for data from the MyConnectome Project and HCP datasets. For each session/subject, a response function was estimated with the Dhollander algorithm, however, as acquisition protocols differed between the two datasets, no average response function was derived. FODs were again estimated with MSMT-CSD, using the individual response functions and followed by FOD normalization. Normalized FODs were transformed and reoriented to the template space.

#### Streamline Tracking and Quantification

Whole-brain probabilistic tractography was performed for the template and analysis datasets with MRtrix3, using the iFOD2 probabilistic algorithm (Tournier et al., 2010) with default parameters. Random seeding of tractography was performed throughout the brain until targets of 100,000 and 10,000,000 streamlines have been selected for the template and analysis datasets, respectively. Tractography was then filtered to fit the amplitudes of the associated FODs using spherical-deconvolution informed filtering of tractograms (SIFT) (Smith et al., 2013) until streamline counts of 50,000 and 1,000,000 remained for template and analysis datasets, respectively. The combination of constrained spherical deconvolution (CSD), iFOD2 generated tractography, and SIFTing has previously been shown to improve tracking of streamlines, particularly in regions of multiple fiber orientations, while preserving tract densities reflective of the underlying diffusion signal.

Tensor images were additionally computed on intensity normalized diffusion weighted images (DWI) of the analysis datasets, which had also been transformed to template space. Diffusion tensor images (DTI) were estimated using an iteratively reweighted linear least squares estimation (Veraart et al., 2013). Fractional anisotropy (FA) measurements were derived from DTI and mapped to corresponding streamlines, enabling further quantitative analysis following clustering.

#### Spectral Clustering (Method 1)

Using spectral clustering (von Luxburg, 2007), bundles of streamlines (tracts) were initially identified on the SIFTed tractography template before propagating cluster labels to tractography from analysis datasets based on similarity of streamline trajectory. First, individual streamline similarity was assessed with comparisons to all other streamlines of the template. Twenty equispaced samples, inclusive of endpoints, were taken along the length of each streamline and a minimum average, direct-flip (MDF) distance was used to compute between corresponding samples across streamlines (Visser et al., 2011; Garyfallidis et al., 2012; Guevara et al., 2012; Siless et al., 2013) and generate a distance matrix. Streamlines whose distances were greater than two standard deviations from the average whole-brain streamline distance were deemed to be outliers and discarded, similar to O’Donnell et al. (2017). An affinity matrix, characterizing similarity between streamlines, was created with the application of a Gaussian kernel with a width of 8 mm to the distance matrix.

Spectral clustering, which has been previously employed in tractography clustering (O’Donnell and Westin, 2007; Siless et al., 2018; Zhang et al., 2018b), utilizes Laplacian matrices as one of the primary tools (von Luxburg, 2007). Following the implementation described by Ng et al. (2002), spectral clustering was performed on the template tractography to label and assign streamlines to a cluster. A selection of *k* = 800 clusters was chosen following qualitative assessment of clusters ranging from *k* = 400 to *k* = 1,400. The qualitative assessment involved visual inspection of identified tracts for each chosen number of clusters and was performed to assess the ability to discern tracts with noticeably different trajectories. This selection of 800 clusters was also determined to be the optimal number of clusters by O’Donnell and Westin (2007), and later employed by Zhang et al. (2018b). Established clusters were colored according to the coordinates of the cluster centroids, as described by Brun et al. (2003).

#### QuickBundles (Method 2)

For comparison, the template tractography was also clustered utilizing QuickBundles (Garyfallidis et al., 2012) before sub-sampling and propagating labels to tractography of analysis datasets as before to establish tract correspondence. Briefly, QuickBundles computes the MDF distance between unassigned streamlines with a centroid streamline from existing clusters, updating the cluster centroid as new streamlines are added. The computed distance is compared against a user-chosen distance threshold and if the distance is within the threshold, it is assigned to the cluster with the smallest distance, otherwise it is assigned as a new cluster. Garyfallidis et al. (2012), utilizing lower thresholds result in more detailed representations of underlying trajectories, while higher thresholds result in the merging of bundles which may have similar trajectories. Additionally, the user can choose to set the maximum number of clusters, such that once the maximum number of clusters is reached, new streamlines are only assigned to existing clusters.

As was done for spectral clustering, streamlines were resampled to 20 equispaced samples in order to compute the MDF distance for QuickBundles, selecting a maximum of *k* = 800 clusters and a distance threshold of 8 mm was chosen to match the number of clusters and kernel width, respectively, from spectral clustering. Following cluster assignment, streamlines were colored using the cluster centroid as was done for spectral clustering.

#### Labeling Analysis Datasets

Both spectrally clustered and QuickBundles clustered methods used a labeled sub-sample containing 20,000 streamlines of the template tractography to assign labels to streamlines identified in the analysis datasets. A sub-sample of the labeled template was required due to computational memory limitations. Streamline similarity between the sub-sampled tractography template and the tractography from analysis datasets was also computed using the MDF method as previously described. Labels from template streamlines were propagated to the tractography from analysis datasets based on maximum similarity, establishing correspondence between the most similar tracts.

#### Short Range, “U”-Shaped Streamlines

Streamlines comprising short-range, “U”-shaped tracts were identified and extracted from whole-brain tractography using adapted parameters (Guevara et al., 2017; O’Halloran et al., 2017) to extract from whole-brain tractography. Identification of “U”-shaped streamlines utilized the Euclidean distance between streamline endpoints (D), computed as the Euclidean distance between the terminal ends of a streamline, and streamline length (L), computed as the arc length of the sample points (s_*i*_).


$$L=\sum_{i=1}^{N}\left|S_{i}-S_{i-1}\right|$$

$$D=\left|S_{N}-S_{1}\right|$$

To extract streamlines with the expected “U”-shaped curvature, the end point distance was constrained to approximately one-third of the streamline length ($D<\frac{L}{\pi }$), as employed by O’Halloran et al. (2017). Additional streamline length constraints of 20 mm (minimum) and 80 mm (maximum) were imposed (20*mm*≤*D*≤80*mm*). Streamlines which crossed across brain hemispheres were removed.

Clustering techniques were separately employed to identify bundles of “U”-shaped streamlines after extraction. Both spectral clustering and QuickBundles were applied, using a 6 mm Gaussian kernel and distance for both techniques (smaller than what was previously employed for whole-brain tractography. For short-range, “U”-shaped tracts, *k* = 500 was determined to be optimal to identify individual “U”-shaped tracts after qualitative assessment of clusters from *k* = 200 to *k* = 700. As before, qualitative assessment involved visual inspection of clusters for the ability to discern unique tract trajectories. Endpoints of identified “U”-shaped tracts were used to identify connectivity between cortical parcellations of the Desikan-Killiany atlas (Desikan et al., 2006), assigning tract endpoints to the nearest parcels within a 4 mm radius (Smith et al., 2015). If a tract was determined to connect multiple parcels (e.g., one end of the tract terminates within two parcels), assignment was determined by maximum streamline count. Cortical parcellations were mapped to lobes by the given approximate Freesurfer mapping (Klein and Tourville, 2012). Streamlines determined to be in the cerebellum were not considered to be part of the superficial white matter of interest and excluded from subsequent analysis.

### Analysis

In the following subsection, we describe the metrics used to assess reliability of template-based tractography clustering. Briefly, we computed the centroid distances between the average dataset centroid and individual subject or session centroid within the respective datasets, compared the voxel-wise spatial overlap of identified tracts, and examined the streamline counts of identified tracts. Analysis was performed on corresponding tracts within each dataset. An unpaired *t*-test was also performed to determine whether there was a difference in the resulting metrics from the two cluster algorithms.

#### Distance From Average Centroid

Tract centroids were computed for all tracts identified in both analysis datasets by averaging spatial components of corresponding sample points across streamlines. A dataset average tract centroid (here-on referred to as the average centroid) was also computed by averaging the centroids computed across the subjects and sessions within the respective analysis datasets. An Euclidean distance was computed for corresponding tracts between the average centroid and centroids from the analysis datasets by employing the MDF distance previously described.

#### Voxel-Wise Spatial Overlap of Tracts

First, a tract density map for each cluster was created by identifying streamline counts passing through each voxel. Then, the fraction of each tract (a value between 0 and 1) passing through a voxel was determined from the tract density map to assess the weighted Dice similarity coefficient (wDSC) (Cousineau et al., 2017b). Briefly, the wDSC is a modified version of the conventional Dice similarity coefficient (Dice, 1945) for assessing overlap of tractography, weighting more heavily the denser regions of a tract instead of penalizing streamlines further from the core as is done by conventional Dice (Cousineau et al., 2017b). The wDSC was computed with the following equation (Eq. 3), where A_*v*_ and B_*v*_ represent the fraction of streamlines passing through a voxel of two corresponding tracts and v’ represents a voxel within the intersection of A and B.


$$w\,D\,S\,C\,\left(A,B\right)=\frac{\sum_{v^{\prime }}A_{v^{\prime }}+\sum_{v^{\prime }}B_{v^{\prime }}}{\sum_{v}A_{v}+\sum_{v}B_{v}}$$

Average wDSC within the analysis datasets were computed across corresponding tracts.

#### Along-Tract Fractional Anisotropy Similarity

To assess reliability of quantitative scalar metrics along identified tracts, intraclass correlation (ICC) of along-tract fractional anisotropy (FA) was computed. Here, FA was chosen due to its widespread use and interpretation in a number of diffusion studies. Cousineau et al. (2017a) previously highlighted the benefits of examining quantitative metrics along the length of a tract for examining reliability . A two-way, random effects model (McGraw and Wong, 1996) was employed to evaluate absolute agreement of FA at corresponding samples along the length of a tract. Utilizing this model, the column factor (“raters”) were the samples along the tract, and the row factor (“targets”) were the individual subjects or sessions of the analysis dataset.

#### Streamline Count and Variation

Streamline counts comprising each tract were extracted and compared across corresponding tracts and the subjects and sessions within the respective analysis datasets. Streamline counts enabled assessment of reconstruction consistency and importantly, may be used to determine tracts which may not be reliably identified. The extent of streamline count variability of each identified tract was also evaluated by computing the coefficient of variation (CV). Here, the CV was calculated as the standard deviation of the streamline count (σ) over the average streamline count (μ) for corresponding tracts within each analysis dataset ($C\,V=\frac{\sigma }{\mu }\times 100\%$).

#### Relationship Between Metrics

To determine whether a relationship existed between the different reliability metrics examined, a Spearman correlation is computed between the described metrics used for reliability analysis. After computing the Spearman correlation between all metrics, false discovery rate correction was performed following the Benjamini-Hochberg procedure (Benjamini and Hochberg, 1995).

## Results

### Distance From Average Centroid

**FIGURE 2:**
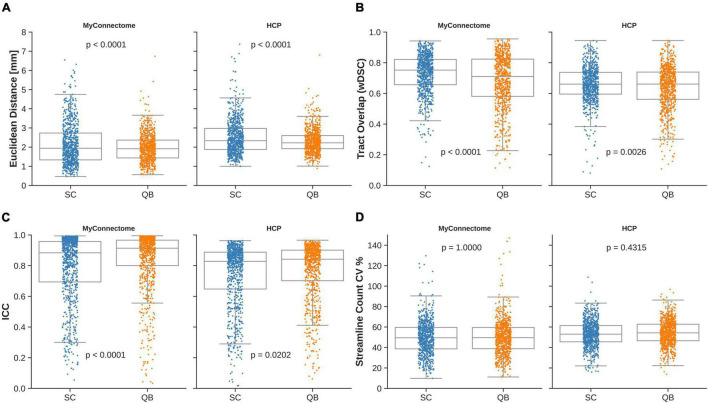
Individual observations for a given metric are overlaid on a box plot for each dataset and clustering method employed by the template. **(A)** Mean Euclidean distance of tracts relative to the corresponding average tract centroid. **(B)** Average voxel-wise spatial overlap across corresponding tracts. **(C)** Along-tract absolute agreement of fractional anisotropy across corresponding tracts. **(D)** Variability of streamline counts across corresponding tracts.

The mean Euclidean distances were observed to be 2.16 ± 1.10 mm and 2.51 ± 0.90 mm for the MyConnectome and HCP datasets, respectively, when compared against the average centroid identified from the spectrally clustered template. From the QuickBundle clustered template, an average Euclidean distance of 1.96 ± 0.73 mm and 2.31 ± 0.62 mm was observed for the MyConnectome and HCP datasets when compared against the average centroid. Across datasets, the average Euclidean distances of tracts to the corresponding average centroid was around two voxels (about 2.5 mm). In both datasets, a difference was observed in the computed Euclidean distance for tracts identified using the two clustering algorithms. Figure 2A displays a boxplot with individual points indicating the observed Euclidean distances for a given tract for each dataset. Distributions of Euclidean distances were similar across datasets, with the MyConnectome dataset exhibiting a lower Euclidean distance against the average centroid for both clustering methods than the HCP dataset. Supplementary Table 1 details the average Euclidean distances and standard deviations for all tracts identified.

### Weighted Voxel-Wise Spatial Overlap

Spatial overlap of identified tracts were computed between corresponding tracts identified with both spectrally clustered and QuickBundles clustering algorithms within the analysis datasets. Overlap within analysis datasets demonstrated good overlap in both datasets with wDSC of 0.729 ± 0.129 and 0.661 ± 0.115 for spectral clustering identified tracts in MyConnectome and HCP analysis datasets, respectively. As with the computed overlaps computed in tracts identified via spectral clustering, QuickBundle clustered identified tracts also demonstrated good overlap in both datasets with an average wDSC of 0.683 ± 0.174 and 0.639 ± 0.141 for MyConnectome and HCP analysis datasets, respectively. A difference in resulting tract-overlaps observed for both datasets when using the two different clustering algorithms. Figure 2B displays for each dataset, a box plot with individual points indicating the observed average overlap for a given tract. Full details of computed wDSCs for identified tracts within analysis datasets are provided in Supplementary Table 1 (see Supplementary Videos 1, 2 for their respective algorithms).

### Along-Tract Fractional Anisotropy Agreement

Intraclass correlation (ICC) was computed for each analysis dataset by comparing the along-tract FA at corresponding samples across subjects and sessions. For tracts identified from the spectral clustered template, good absolute agreement was observed with computed average ICCs of 0.792 ± 0.218 and 0.742 ± 0.207 for the MyConnectome and HCP analysis datasets, respectively. The QuickBundle clustered template also demonstrated high agreement of along-tract FA, with average ICCs of 0.841 ± 0.190 and 0.769 ± 0.190 in the MyConnectome and HCP datasets, respectively. For both datasets, ICCs demonstrated a difference when using the two different clustering algorithms. However, with both clustering algorithms, the MyConnectome dataset demonstrated better along-tract agreement. Figure 2C displays for each dataset, a box plot with individual points indicating the observed along-tract FA agreement for a given tract. Supplementary Table 1 provides full details of computed ICC for all tracts, including 95% confidence intervals.

### Streamline Count and Variation

Within the analysis datasets, streamline counts were determined for each subject or session and averaged across corresponding tracts. Tracts lacking streamlines for at least one subject or session of the analysis datasets were identified. Thirteen of 800 (spectral clustering) and 5 of 800 (QuickBundles) tracts of the MyConnectome dataset contained no streamlines across the available sessions, while all tracts of the HCP dataset contained at least a single streamline for the analyzed subjects. No difference in streamline count variability was observed between the two algorithms for either dataset. Figure 2D displays for each dataset, a box plot with individual points indicating the observed streamline count variance of a given tract. Full details regarding streamline counts for each tract and associated dataset information can be found in Supplementary Table 1.

Furthermore, the extent of the variability for each of the identified tracts were examined. The tracts identified via spectral clustering in the MyConnectome dataset exhibited lower average variability (50%) compared against the HCP dataset (53%). However, the range of the variability exhibited was smaller in the HCP dataset (16–109%) than in the MyConnectome dataset (10–203%). Similarly, tracts identified via QuickBundles demonstrated lower variability (50%) in MyConnectome than in HCP (54%), but again showed a smaller range of variability in HCP (14–97%) than in MyConnectome (11–156%). A full summary of average streamline counts and CV can be found in Supplementary Table 1.

### Relationships Between Reliability Metrics

**FIGURE 3:**
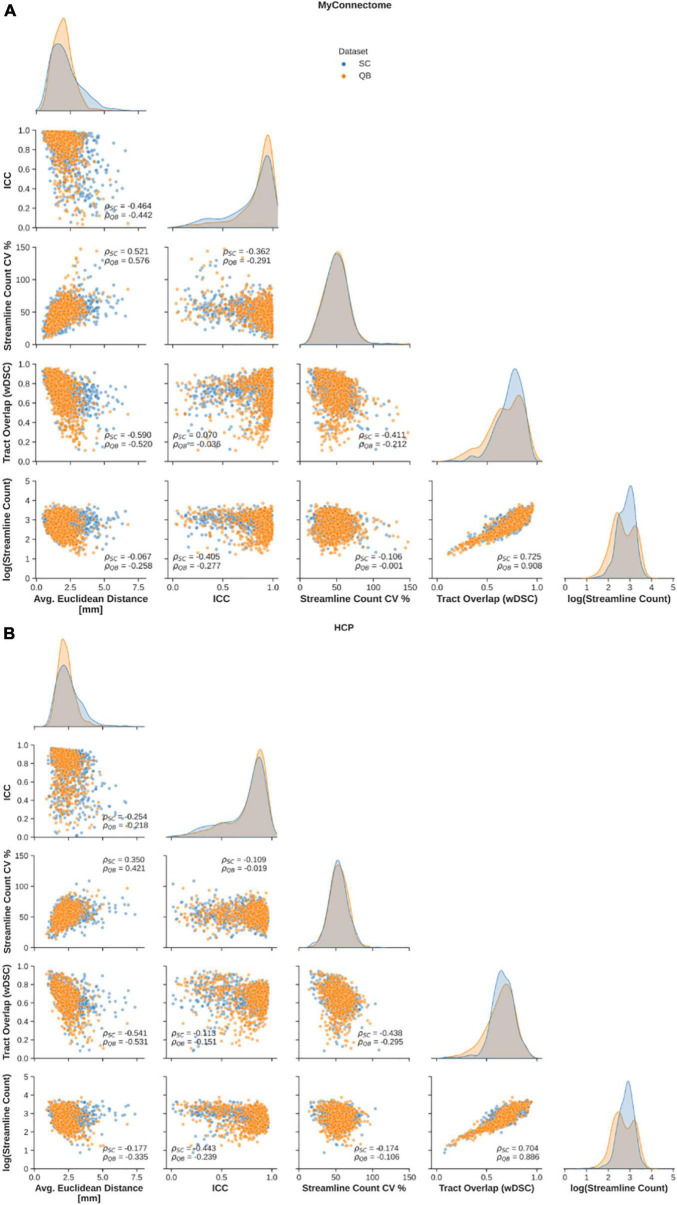
Spearman correlations are computed to explore relationships of metrics employed to assess reliability of spectral clustered (blue circles) and QuickBundle clustered (orange circles) identified tracts via whole-brain tractography. Relationships between different metrics used for assessment are shown in pairplots for **(A)** MyConnectome and **(B)** HCP datasets. Relationships between various metrics and average Euclidean distance from an average tract centroid (left-most), relationships with along-tract absolute agreement (ICC; middle-left column), relationships with streamline count variability (middle-column), and with voxel-wise spatial overlap (middle-right column) are displayed. Distribution of observed points for a given metric (matching the x-axis) are plotted along the diagonal.

Relationships between employed metrics were explored to examine common features in reliable tracts. First, a significant negative correlation was observed between the average Euclidean distance from the average centroid and tract overlap, streamline count variability, while a significant positive relationship was observed between Euclidean distance and streamline count variability (Figure 3, left column). Further, a significant negative correlation was identified between the along-tract agreement of fractional anisotropy and both streamline count variability (only for the MyConnectome dataset) and the average log-transformed streamline count (Figure 3, middle-left column). Finally a significant negative relationship was observed between the tract spatial overlap and streamline count variability (Figure 3, middle column), while a positive relationship was observed between tract overlap and the average log-transformed streamline count (Figure 3, middle-right column). No significant relationship was identified between the tract spatial overlap and the along-tract agreement or between the log-transformed streamline count and average Euclidean distance. Additionally, no significant relationship was identified between streamline count variability and tract overlap in the HCP dataset. Relationships were similar for both tracts identified via spectral clustering and the QuickBundles algorithm. For the majority of identified relationships, the correlation was stronger in the single-subject MyConnectome dataset than in HCP datasets for both algorithms.

### “U”-Shaped Tract Reliability

**FIGURE 4:**
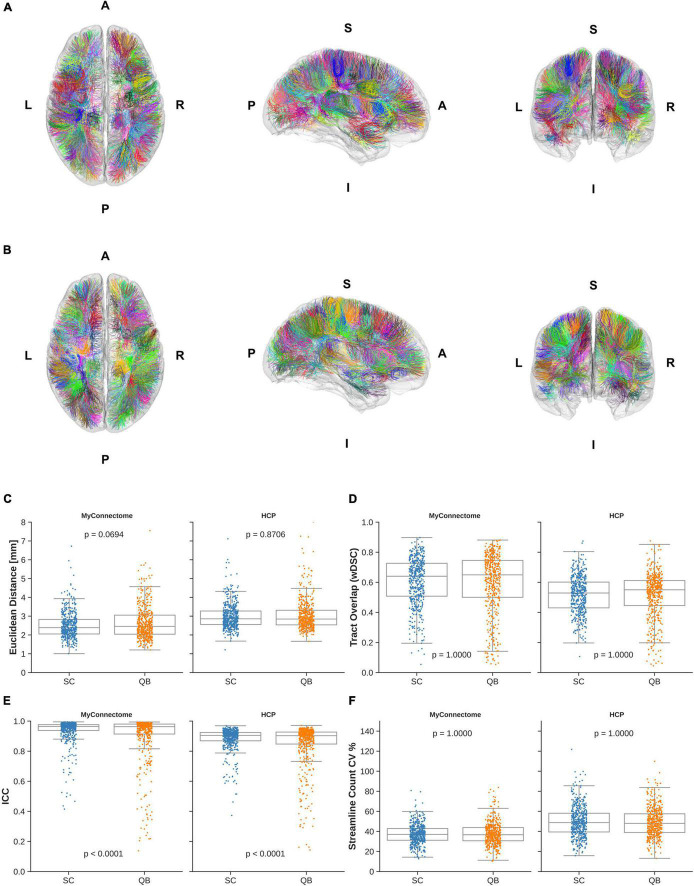
“U”-shaped tracts identified in the HCPUR100 template viewed from axial superior (left), sagittal right (middle), and coronal anterior (right) via **(A)** spectral clustering and **(B)** QuickBundle clustering. Colors of identified tracts do not correspond across clustering methods. Individual observations in “U”-shaped tracts for a given metric are overlaid on a box plot for each dataset and clustering method employed by the template. **(C)** Mean Euclidean distance of tracts relative to the corresponding average tract centroid. **(D)** Average voxel-wise spatial overlap across corresponding tracts. **(E)** Along-tract absolute agreement of fractional anisotropy across corresponding tracts. **(F)** Variability of streamline counts across corresponding tracts.

Assessment of short-range, “U”-shaped tracts was performed with the same metrics used to examine reliability of whole-brain tractography. The average Euclidean distance from the average centroid for identified “U”-shaped tracts via the spectrally clustered template was similar as previously observed, with distances of 2.53 ± 0.75 mm and 2.99 ± 0.67 mm in MyConnectome and HCP datasets, respectively. From the QuickBundle clustered template, a slightly greater distance is observed—2.66 ± 0.92 mm and 3.05 ± 0.82 mm for MyConnectome and HCP datasets, respectively. Figures 4A,B display the identified tracts from spectral clustering and QuickBundles, respectively (see Supplementary Videos 3, 4 for individually identified tracts for the respective algorithms), while Figure 4C displays a box plot with individual Euclidean distance observations against the average tract centroid for each dataset.

Spatial overlap of identified “U”-shaped tracts from spectral clustering and QuickBundles were also computed. Overlap within analysis datasets demonstrated moderate overlap in both datasets with wDSC of 0.606 ± 0.155 and 0.517 ± 0.123 for spectral clustering identified tracts in MyConnectome and HCP analysis datasets, respectively. With QuickBundle clustered identified tracts, similar overlaps were observed with an average wDSC of 0.598 ± 0.199 and 0.515 ± 0.152 for MyConnectome and HCP analysis datasets, respectively. Full details of computed wDSCs for identified “U”-shaped tracts compared for the analysis datasets are provided in Supplementary Table 2.

As with whole-brain tractography reliability, the absolute agreement of along-tract FA was also computed for “U”-shaped tracts identified in the analysis datasets, comparing the metrics mapped at corresponding samples across subjects and sessions. For tracts identified from the spectral clustered template, good absolute agreement was observed with computed average ICCs of 0.938 ± 0.081 and 0.883 ± 0.076 for the MyConnectome and HCP analysis datasets, respectively. The QuickBundle clustered template also demonstrated high agreement of along-tract FA, with average ICCs of 0.900 ± 0.162 and 0.847 ± 0.147 in the MyConnectome and HCP datasets, respectively. Similar to whole-brain clustering, a difference was observed between the two clustering algorithms applied to both datasets. As before, the MyConnectome dataset demonstrated better along-tract agreement irrespective of the clustering method applied to the template. Supplementary Table 2 provides full details of computed ICC for all “U”-shaped tracts, with 95% confidence intervals.

Similarly, as previously observed, not all analysis datasets contained streamlines for all template identified tracts. Six6 tracts in both the MyConnectome and HCP datasets contained no streamlines when tracts were identified with the spectrally clustered template, while 32 and 24 tracts, respectively, was found to contain no streamlines when identified with the QuickBundle clustered template. Variability of tract streamline counts was also comparable, ranging from 13 to 81% and 16–122% (averaging 37 and 50%) for MyConnectome and HCP datasets, respectively, identified via the spectrally clustered template. Similarly, variability of identified tract streamline counts from the QuickBundle clustered template ranged from 11 to 84% and 14–110% (averaging 38 and 49%) for MyConnectome and HCP datasets. Figure 4 displays for each dataset, a box plot with individual points indicating the observed values of a given tract for each described metric. A full summary of evaluated metrics of short-range, “U”-shaped tracts, inclusive of streamline counts can be found in Supplementary Table 2.

**FIGURE 5:**
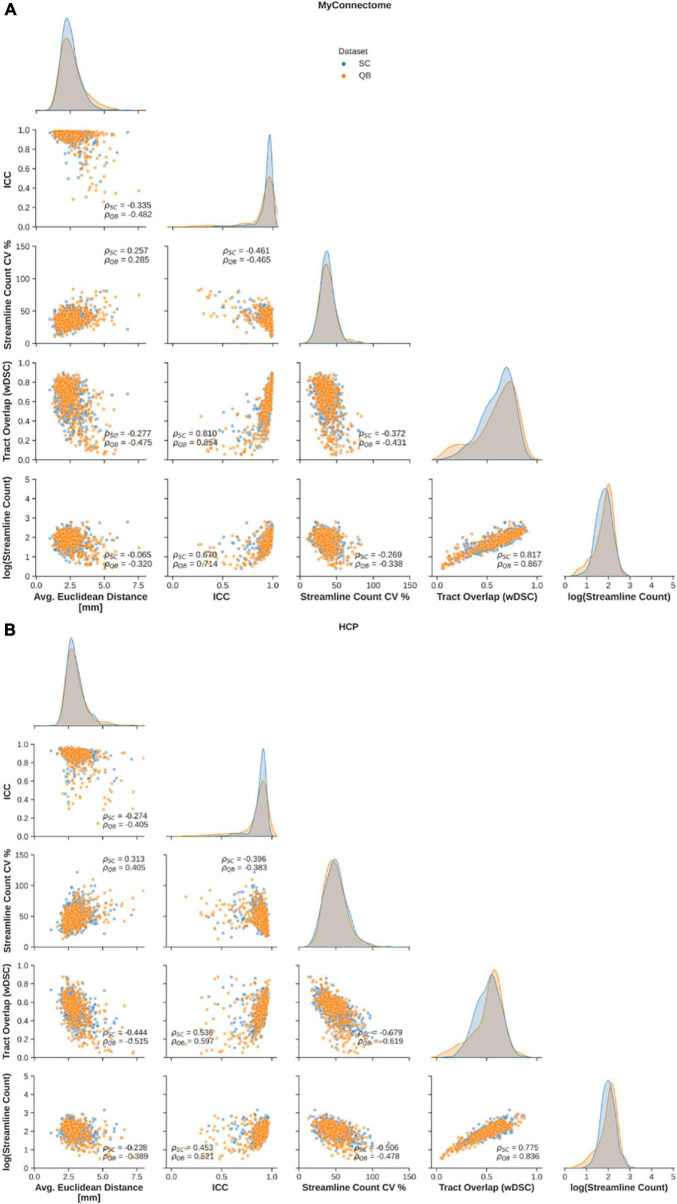
Spearman correlations are computed to explore relationships of metrics employed to assess reliability of spectral clustered (blue circles) and QuickBundle clustered (orange circles) identified tracts in short-ranged, “U”-shaped tracts. Relationships between different metrics used for assessment are shown in pairplots for **(A)** MyConnectome and **(B)** HCP datasets. Relationships between various metrics and average Euclidean distance from an average tract centroid (left-most), relationships with along-tract absolute agreement (ICC; middle-left column), relationships with streamline count variability (middle-column) and with voxel-wise spatial overlap (middle-right column) are displayed. Distribution of observed points for a given metric (matching the x-axis) are plotted along the diagonal.

The relationships between different reliability metrics were also similar to the relationships observed for whole-brain tractography clustering. Negative correlations were observed with the Euclidean distance were demonstrated for all metrics except for streamline count variability, which exhibited a positive relationship (Figure 5, left-most column). As with whole-brain tractography, negative correlations were identified between along-tract agreement of fractional anisotropy and both streamline count variability and the average log-transformed streamline count (Figure 5, middle-left column). Lastly, a significant positive relationship was once again identified between the log-transformed streamline count (Figure 5, middle column) and voxel-wise spatial overlap (Figure 5, middle-right column). As before, the relationships were similar for “U”-shaped tracts identified via spectral clustering and the QuickBundles algorithm.

Lobular connectivity of “U”-shaped tracts was identified and summarized. The majority of tracts identified in both hemispheres were found within the frontal lobes, followed by the parietal lobes. A number of tracts were also identified to connect between the frontal and parietal lobes. A full summary of the lobular connectivity of “U”-shaped tracts can be found in Supplementary Table 3.

## Discussion

### Clustering Reliability

Reliable identification of white matter pathways is crucial for increasing confidence in the subsequent analysis. In this work, we investigated the reliability of template-based clustering by identifying and evaluating metrics of reliability in identified tracts. On average, we observed identified tracts to exhibit a Euclidean distance around 2.5 mm (or two voxels) from the average centroid. A deviation from the average tract trajectory could result in an increasing Euclidean distance. Other factors, such as dispersion of streamlines (e.g., fanning in the corticospinal tract), could also contribute to an increased Euclidean distance.

Another reliability metric evaluated was the voxel-wise spatial overlap of corresponding tracts. Previous studies have used a Dice similarity coefficient to compute tract overlap (Schilling et al., 2019; Rheault et al., 2020), but wDSC was chosen as it better reflects the overlap of streamlines by minimizing the penalization of those far from the core (Cousineau et al., 2017b). In healthy individuals, corresponding tracts are generally found in similar regions of the brain with comparable trajectories (more variability is expected in the superficial white matter). The wDSC reflects this similarity by comparing and identifying the voxels traversed by the two tracts being compared. If two tracts have similar trajectories, presumably also traversing similar voxels, this is reflected by a higher degree of spatial overlap when brought into the same space (e.g., template space). In the study by Zhang et al. (2019), clustering demonstrated greater reliability than ROI-based techniques in a study of test-retest datasets, exhibiting a minimum tract overlap of 0.593 from a clustering approach compared to 0.362 with a ROI-based approach. Here, we provide further support for template-based clustering approaches, demonstrating an average wDSC across the two techniques and datasets evaluated that is greater than reported by Zhang et al. (2019) indicating a high degree of overlap.

Along-tract quantitative measurements can also be a good indicator of tract reliability, as noted by Cousineau et al. (2017a). Corresponding tracts are expected to have similar along-tract profiles and deviation from this profile could indicate an incorrect tract was identified. In this study, the absolute agreement of along-tract fractional anisotropy was assessed between corresponding samples of tracts across subjects and sessions. The majority of identified tracts within a given dataset and technique exhibited good agreement between their tract profiles.

Tract streamline counts and the variability across subjects and sessions were also evaluated. While the method of tractography seeding can influence the resulting streamlines, methods such as SIFT, were developed to filter and retain streamlines such that streamline counts are reflective of the underlying diffusion profile. Further, different individuals may also contribute to this variability due to underlying anatomy. However, within a single healthy adult individual with developed brain, tract streamline counts should be similar (i.e., on the same order of magnitude). In this study, a slightly smaller variability was observed in the MyConnectome dataset relative to the HCP dataset, but a high variability was still demonstrated in the majority of identified tracts. The observed variability could suggest streamline counts and the associated variability may not be a dependable indicator of reliability. Nonetheless, if streamline counts are to be assessed, care processing should be performed to ensure they are comparable, such as applying post processing techniques to correspond to the underlying diffusion signals, such as with SIFT (Smith et al., 2013).

### Reliability Metric Relationships

While the chosen metrics evaluated can all be used individually to characterize the reliability of tract identification, certain relationships were observed between different metrics of reliability. We have shown that the spatial overlap of identified tracts demonstrated good reliability across other metrics, such as low streamline count variability demonstrating the need for filters like SIFT that attempt to match the streamline counts to underlying diffusion signals. Similarly, a low Euclidean distance was also observed with low streamline count variability, as well as high tract overlap. However, along-tract agreement (ICC), did not demonstrate such a relationship with other reliability metrics. Instead both high and low reliability across other metrics were observed when ICC suggested good reliability. Lastly, similar relationships were observed for both whole-brain tractography and “U”-shaped tractography, suggesting that template-based clustering may be appropriate for both.

### “U”-Shaped Tract Clustering

Clusters identified from whole-brain tractography can contain multiple “U”-shaped tracts clustered together and affecting the evaluated metrics described previously. One such example was in clusters with a large number of streamlines (>1,000). The close proximity of these streamlines could contribute to a smaller Euclidean distance and a high degree of overlap observed. As such, it is important to separate evaluation of “U”-shaped tracts from whole-brain tractography, which is possible by lowering the Gaussian kernel width for spectral clustering and the distance threshold for QuickBundles.

We separately assessed the clustered “U”-shaped tracts with the same metrics used to study whole-brain tractography. Much of the same observations noted previously in whole-brain tractography were also seen in these “U”-shaped tracts. The use of a template may alleviate some of these issues, capturing tracts that have similar trajectories across individuals. Conversely, “U”-shaped tracts specific to an individual may be missed. Notably, a slight increase in the computed Euclidean distance was observed for QuickBundle identified tracts. Further, a decrease in spatial overlap was observed in “U”-shaped tracts irrespective of the algorithm chosen. Additionally, the QuickBundles clustered template resulted in the lack of streamlines in more tracts of both datasets, which could be due to clusters of outlier streamlines in the template. It has been previously noted that the QuickBundles method may capture outlier streamlines in small clusters (Siless et al., 2013) as it uses a distance threshold for cluster assignment without discarding any streamlines. While discrepancies observed between clustering algorithms may be attributed to implementation differences of evaluated algorithms or the choice of parameters, an overall decrease in reliability (as seen from tract overlap), indicates that improvements still need to be made to improve the reliability of identifying “U”-shaped tracts.

### Inter- vs. Intrasubject

In this work, we utilized two unique datasets: an intersubject dataset acquired to investigate the human brain in the HCP and an intrasubject dataset acquired over a 3-year period to similarly investigate the human brain using similar acquisitions. In assessing template-based clustering reliability, similar observations were made across both datasets. While minimal change would be expected in the developed brain of a single subject, some variation is expected across different individuals (Cousineau et al., 2017b), which may contribute to the differences observed between the two datasets. When examining “U”-shaped tracts, this expectation appeared to be reflected in the evaluated metrics, with tracts identified in the single subject dataset demonstrating slightly less variability as previously noted.

### Template-Based Clustering

In addition to the use of two unique datasets, the processing was also performed with two different clustering tools using similar parameters: spectral clustering and QuickBundles (Garyfallidis et al., 2012). Analysis was performed on both for comparison of reliability in two different template-based clustering tools. Differences between the two clustering algorithms were observed, particularly when performing whole-brain clustering. These differences may be attributed to disparities in the implementation of the two algorithms, including the handling of outlier streamlines as previously mentioned. Further, while the clusters may not correspond across between these two different techniques, and the relationships observed from the results of both techniques were similar, suggesting the robustness of a template-based approach in reliably identifying tracts. A previous study had explored the challenges of tractography, assessing the pathways identified by various different methods (Maier-Hein et al., 2017). Here, we explored the reliability of template-based clustering algorithms. As tools and techniques are developed and refined to automate tract identification, the importance of assessing the reliability of these methods should be emphasized.

### Limitations

Clustering of tractography, both with spectral clustering and using QuickBundles, required streamlines to be resampled to N equispaced samples. Subsequent analysis was also performed on these samples along a given tract. However, streamlines comprising a tract may be of different lengths, with some streamlines terminating earlier than others due to meeting cutoff criteria. Despite differing lengths, correspondence is assumed between two samples. One method of resolving this is to set terminal ROIs at the ends of a tract such that all streamlines are guaranteed to terminate or be cutoff at the ROIs. As previously noted when discussing manual placement of ROIs, this requires some anatomical knowledge (Tunç et al., 2014). Alternatively, Chandio et al. (2020) mapped samples from streamlines to a corresponding segment of a representative centroid. This eliminates the need for ROIs, but still requires an adequate registration. Further evaluation of this method is also required to determine its accuracy in mapping superficial white matter.

Clustering performed in this study also used a template to identify corresponding tracts in the analysis datasets. While clustering does not explicitly require registration, the template-based techniques examined here require an adequate registration between the template and the subject of interest (Yendiki et al., 2011; Guevara et al., 2012; Tunç et al., 2014; d’Albis et al., 2018) to identify corresponding tracts across subjects and sessions. Additionally, template-based techniques can only identify tracts with similar trajectories to those already defined by the template. ROI-based techniques can be used to identify tracts of interest, but as mentioned in the introduction, these methods can be laborious and require anatomical knowledge. A combination of the data-driven approaches taken here complemented by the use of ROIs for refinement to ensure proper termination of pathways may be better suited to aid discovery of new tracts *in vivo*.

As previously mentioned, the clustering performed utilized two different techniques and comparison of tracts across these two methods was not possible due to lack of correspondence of identified tracts. Differences include how streamlines were clustered, where spectral clustering performs k-means clustering in a spectral space to identify tracts, QuickBundles identifies similar streamlines by directly employing the MDF distance and adding the streamline to a cluster if the distance threshold is satisfied. Differences in the algorithm are likely the cause behind the differences observed. Nonetheless, the metrics used to evaluate the identified tracts and the comparison of the metrics can be applied generally to assess reliability regardless of the tract identification technique chosen.

Despite the influence of the HCP acquisition protocol on the MyConnectome acquisition protocol, there are notable differences between the two datasets. HCP dMRI was acquired with 3-shells and 270 total directions (90 directions/shell), while MyConnectome dMRI was acquired with 2-shells and 60 total directions (30 directions/shell). Additionally, preprocessing of data may slightly differ, with the HCP data preprocessed with the HCP minimal preprocessing pipeline (Glasser et al., 2013) and the MyConnectome data preprocessed using an in-house pipeline to apply standard preprocessing steps. Preprocessed HCP dMRI data was also corrected for gradient field inhomogeneities, whereas the correction for gradient field inhomogeneities was not possible for the MyConnectome dataset due to the lack of a proprietary scanner-specific file required. Data harmonization—an active area of research—is one possible method to improve comparability between different datasets, as such, future work should also explore the reliability of harmonized datasets.

To match the number of diffusion acquisitions available in the MyConnectome dataset, *n* = 15 subjects were selected from the HCP dataset to keep analysis as similar as possible between the two datasets. As previously mentioned, variability within a single, healthy adult individual is expected to be minimal, there may be more variability in the larger population. In particular, more variability may be expected in the superficial white matter due to differing cortical folding patterns across individuals. In this study, we have shown the ability of template-based clustering to identify corresponding tracts across individuals in the limited sample size. Future studies should explore and quantify the amount of variability, in particular to the superficial white matter, across a larger population.

With regards to clinical applications, such as in neurological and psychiatric disorders, which have been recognized as disorders of the network (e.g., epilepsy as a network disorder (Mark et al., 2012), abnormal networks in schizophrenia (Rubinov and Bullmore, 2013), and more), the capability to identify connectivity throughout the brain, including previously unnamed and unidentified tracts in a reliable manner has important clinical implications. The techniques applied in this study may be able to provide biomarkers indicative pathological changes if tracts can be identified in the presence of disease. However, one of the current limitations of the template-based approach is the requirement of adequate registration, which may be non-trivial with the occurrence of substantial morphological change (e.g., due to tumors). The current study suggests that while template-based approaches are reliable for identifying connectivity and may be a critical approach in expanding current knowledge of the human connectome with potential future clinical impact, improvements are required to tackle the challenges of identifying connectivity in the presence of disease.

## Conclusion

In this work, we performed whole-brain tractography on two unique datasets, assessing the reliability of template-based clustering approaches and identifying relationships between reliability metrics. Similar relationships were observed irrespective of the clustering algorithm chosen suggestive of the robustness of template-based approaches. Furthermore, streamline count on its own may not be a good indicator of reliability, though the evaluation of the metric relationships suggest that the certain metrics may be in agreement with other measures, providing a better indicator of reliability. We further identified the superficial white matter (“U”-shaped) tracts using the same clustering algorithms to assess reliability of a template-based approach, observing similar relationships as in whole-brain tractography. Data-driven, template-based approaches can reliably identify and investigate pathways, including those previously unnamed or unidentified such as the superficial white matter.

Future work should look to examine reliably identified “U”-shaped tracts to improve understanding of its biophysical properties, the relationship with cortical measurements (e.g., gyrification), and how the short-range pathways are affected in patient populations.

## Data Availability Statement

The original contributions presented in the study are included in the following Federated Research Data Repository (FRDR; doi: 10.20383/102.0383 at time of publication) and Supplementary Material.

## Ethics Statement

The studies involving human participants were reviewed and approved by the University of Western Ontario Health Sciences Research Ethics Board (UWO HSREB) under protocol #108456 (PI: Khan). The patients/participants provided their written informed consent to participate in this study.

## Author Contributions

JK: data processing and analysis. AK: project conception and supervision. Both authors contributed to the article.

## Conflict of Interest

The authors declare that the research was conducted in the absence of any commercial or financial relationships that could be construed as a potential conflict of interest.

## Publisher’s Note

All claims expressed in this article are solely those of the authors and do not necessarily represent those of their affiliated organizations, or those of the publisher, the editors and the reviewers. Any product that may be evaluated in this article, or claim that may be made by its manufacturer, is not guaranteed or endorsed by the publisher.

## Footnotes

1
www.github.com/khanlab/mrtpipelines

2
www.github.com/khanlab/neurobeer

3
https://humanconnectome.org/study/hcp-young-adult/document/1200-subjects-data-release

4
http://myconnectome.org/wp/53-2
